# Vascular Forum: collective intelligence in the resolution of vascular clinical cases

**DOI:** 10.1590/1677-5449.005018

**Published:** 2018

**Authors:** Fabiano Luiz Erzinger, Walter Junior Boim de Araujo, Amanda Ayako Minemura Ordinola, André Felipe Gasparini, André Vítor Timóteo da Luz, Daniela Midori Kamada, Alexandre Campos Moraes Amato

**Affiliations:** 1 Instituto da Circulação, Curitiba, PR, Brasil.; 2 Universidade Santo Amaro – UNISA, São Paulo, SP, Brasil.

**Keywords:** intelligence, discussion forums, medical informatics, medical education

## Abstract

**Background:**

Collective intelligence is extremely important in collective groups that discuss clinical medical cases, assisting professionals in their decision-making processes, and consequently, helping their patients.

**Objectives:**

To evaluate the rate of resolution and characteristics of the clinical discussions carried out in a private instant messaging group and its clinical applicability.

**Methods:**

Retrospective analysis of clinical discussions and events on the Vascular Forum, an open group for specialists accessed with mobile devices.

**Results:**

From July 2015 to July 2017, 1013 subjects were discussed among 680 members. Twenty-six (2.57%) of these subjects were curiosities, 101 (9.97%) related to diagnostic doubts, 492 (48.57%) to treatment doubts, and 205 (20.24%) to general doubts, while 189 (17.5%) were case reports. The mean number of interactions per event was 16.599 (±1.366). The mean time from posting of a new subject to the first reply was 42.14 (±7.55) min. The subject discussed was miscellaneous in 358 cases, venous in 336 cases, lymphatic in 15 cases, and arterial in 304 cases and the total number of replies was 15985.

**Conclusions:**

Interaction between experts using instant messaging technology proved capable of raising subjects for discussion and eliciting management approaches quickly. The rate of resolution, defined as the time taken for the first correct answer to be posted, was also high. The Vascular Forum proved to be a tool of great clinical value for its participants, confirming the importance of collective intelligence in medicine.

## INTRODUCTION

 The underlying principles of the concept of collective intelligence are recognition that all human beings have some knowledge, but not all of it, and consideration for their experiences accumulated throughout life, which can be shared. In the context of healthcare, this concept acquires great importance in collective groups for solving clinical medical cases, helping physicians to arrive at decisions promptly and, as a consequence, helping their patients who are dependent on medical excellence. 1

 Since the 1990s, 2 ^,^ 3 medical specialty groups have been created and become popular, employing the same social media and instant messaging technologies that are widely used for day-to-day purposes. Exchange of experiences and ease of access to information have proven extremely valuable, particularly in settings that are far from large centers of information. 

 Formal telemedicine in Brazil has gone through considerable evolution and consolidation over recent years, 4 but advances in technologies and their popularization have enabled informal initiatives, such as collaborative learning networks, to grow. These networks use interactive technologies to expand the possibilities for construction of knowledge, whether by increasing facilities for access to good-quality educational materials, by enabling access to centers of excellence and skilled and experienced colleagues, or through construction of new educational systems. 4

 Among initiatives to promote continuing medical education for health professionals designed to enhance professional qualification, interactive tele education can be an important resource for making high-quality educational materials and modules available. Although a considerable part of educational development is focused on courses, integration into professional practice is fundamental for motivating health professionals. As such, use of methods involving formative specialist second opinions can be important, since they enable educational strategies to be developed with a focus on real-life problem-based learning. 3

 After an informal group of specialists was set up on a secure instant messaging platform, it proved necessary to expand the initiative to create additional subject groups that could cater for more specialists who were interested. Previous experience 2 with other communication media had shown that technology adoption was rapid and there was widespread willingness to participate, enabling productive discussion of controversial subjects, despite the physical distance between participants, in groups comprising fewer than 24 participants. Rapid growth of these groups and constant information exchange created a need to quantify the quality of this initiative and its impact on professional practice. 5 To date, there are no evaluations of vascular groups on instant messaging apps in the literature. 

 The objective of this study is to evaluate the efficacy and efficiency for analysis of clinical cases of an instant-messaging discussion group, in terms of time taken to respond and number of responses posted. 

## METHODS

 A retrospective observational survey was conducted of cases discussed between vascular surgeons in an open instant messaging discussion group with 680 members, over a period spanning from July 2015 to July 2017. The project was approved by the Ethics Committee at the institution, under protocol number 00012017. 

 Forum data are shared between members of the clinical case group using an instant messaging platform (WhatsApp), and were extracted for this study using a specialized application (WhatsApp Pocket), 6 then exported and converted into normal document format. The data were then analyzed and tabulated in a secure online collaborative spreadsheet (GoogleDocs®). 

 The following data from each clinical case were extracted and stratified: physician who initiated the discussion, subject, date, start time, time of first correct response, number of responses, characteristics of the discussion (treatment doubts, diagnostic doubts, curiosities, general doubts, case report), characteristics of the report (summary), presence of extra information in the report (images, test results, texts, and treatment), and definitive diagnosis. The correct response was defined as that with the greatest consensus between participants in the discussion. 

 After manual verification of the consistency of the data, descriptive and statistical analyses were conducted. Characteristics recorded as categorical data were expressed as absolute and proportional frequencies. Statistical analyses employed the *t* test, the chi-square test, and analysis of variance (ANOVA). Multivariate statistical analysis of data was conducted using Excel (Microsoft) and Wizard 1.9.20 (Evan Miller). Characteristics recorded as quantitative data were expressed as means, with standard deviations and ranges. 7

## RESULTS

**Figure 1:**
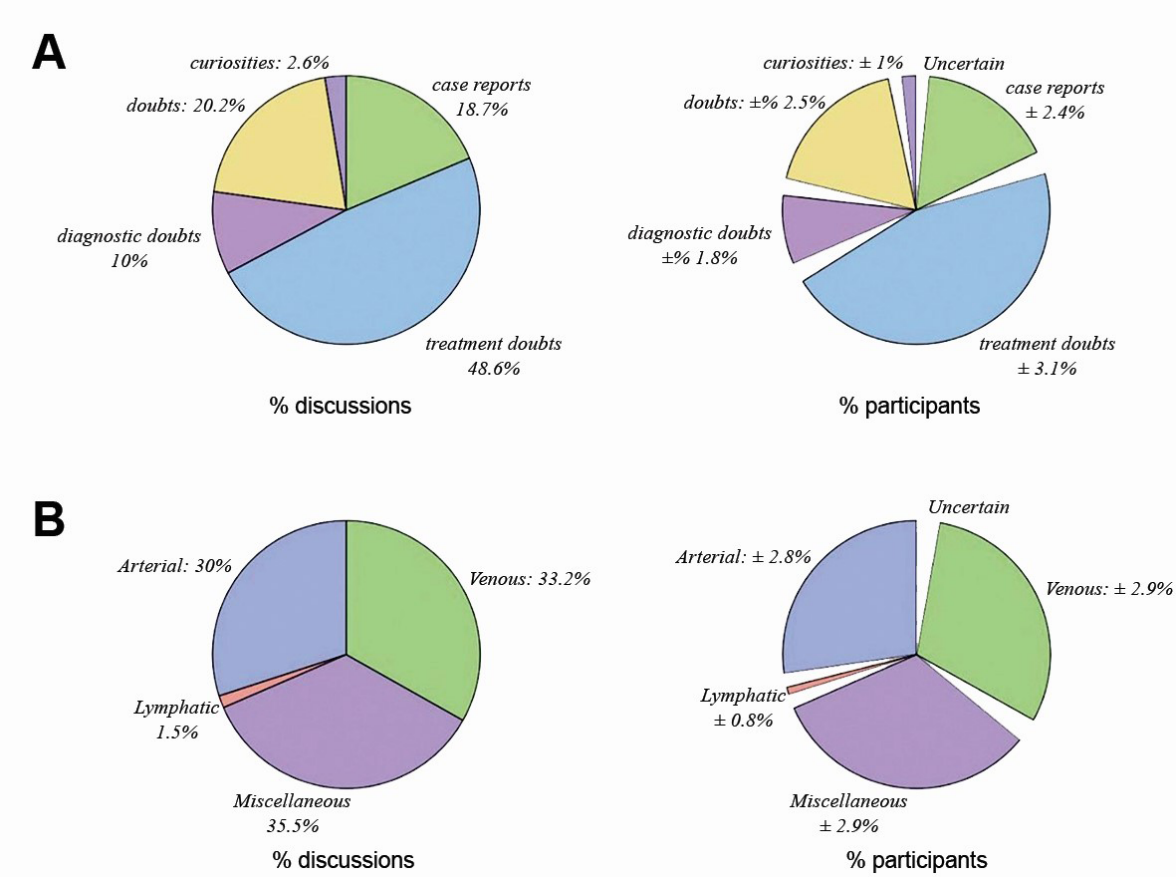
Distribution of discussion characteristics (A) and subjects (B).

**Figure 2:**
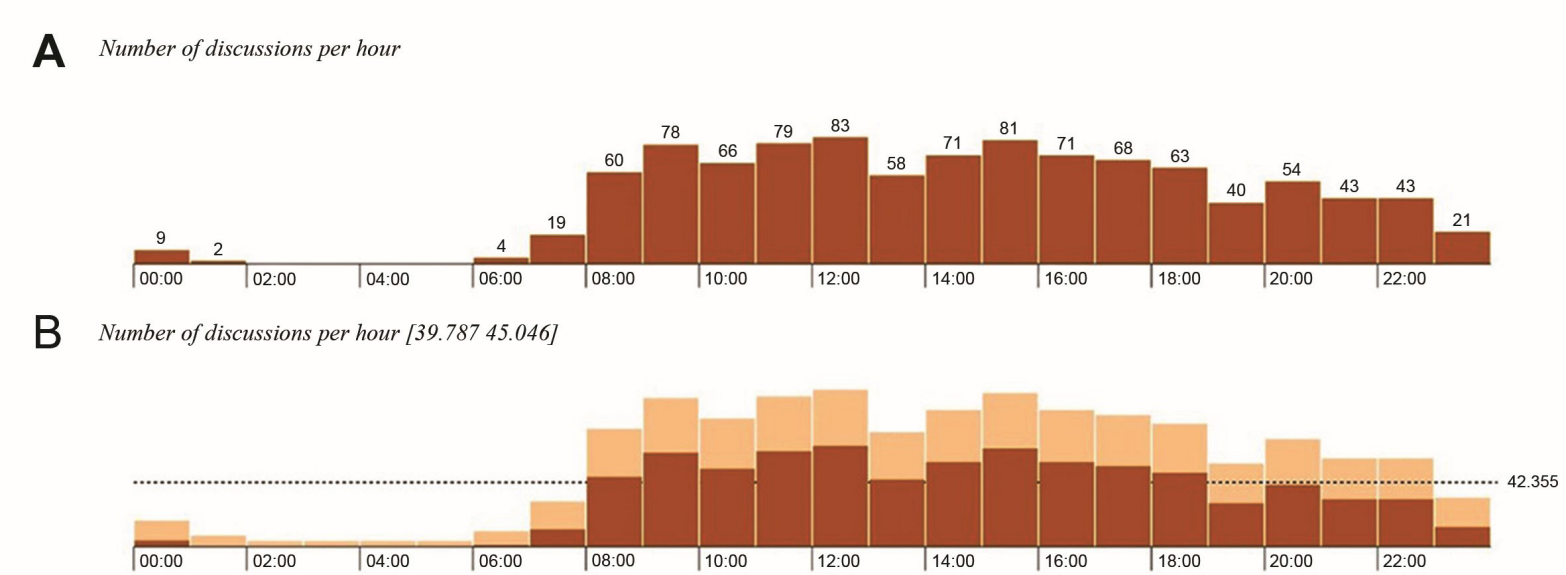
Number of discussions by time of day (A) and replies posted by time of day (B).

 From July 2015 to July 2017, a total of 1,013 different topics were discussed in the instant messaging group. Twenty-six (2.57%) discussions were about curiosities, 101 (9.97%) were about diagnostic doubts, 492 (48.57%) were about treatment doubts, 205 (20.24%) were about general doubts, and 189 (17.5%) were case reports ( Figure 1 ). The mean number of interactions per discussion was 16.6 ± 1.37. The mean time taken for the first reply to be posted was 42.14 ± 7.55 min, while the mean time for the first correct reply, defined as the reply with greatest consensus, was 193.21 ± 19.73 min. Of these discussions, 385 (38%) included photos, 353 (34.8%) included test or examination results, and 426 (42.1%) included treatment details. With relation to time of posting, the group was most active from 8 am to 10 pm ( Figure 2 ). 

 When the discussions were classified by subject, the largest category was miscellaneous, with 358 cases (35.3%), and 5.15 ± 0.47 group members participated. Venous subjects were covered in 336 reports (33.2%), and 5.86 ± 0.45 members participated. Lymphatic subjects were covered in 15 cases (1.5%), and 5.43 ± 1.58 members participated. Arterial subjects were covered in 304 cases (30%), and 5.19 ± 0.44 members participated (ANOVA, p = 0.1133). 

 Assessing the numbers of participants in each discussion by the characteristics of the discussions, 5.56 ± 0.75 group members took part in discussions on general doubts, 4.87 ± 0.57 in case reports, 3.72 ± 1.92 in curiosities, 5.41 ± 0.71 in diagnostic doubts, and an average of 5.61 ± 0.64 group members were involved in each discussion on treatment doubts (ANOVA, p = 0.0728). When analyzed by total number of responses, it was found that discussions about diagnostic doubts had 13.979 ± 2.586 replies, treatment doubts had 17.06 ± 1.70 replies, curiosities had 6.88 ± 3.65 replies, general doubts had 19.98 ± 4.28 replies, and case reports received 14.45 ± 3.27 replies (ANOVA, p = 0.0103). 

**Figure 3:**
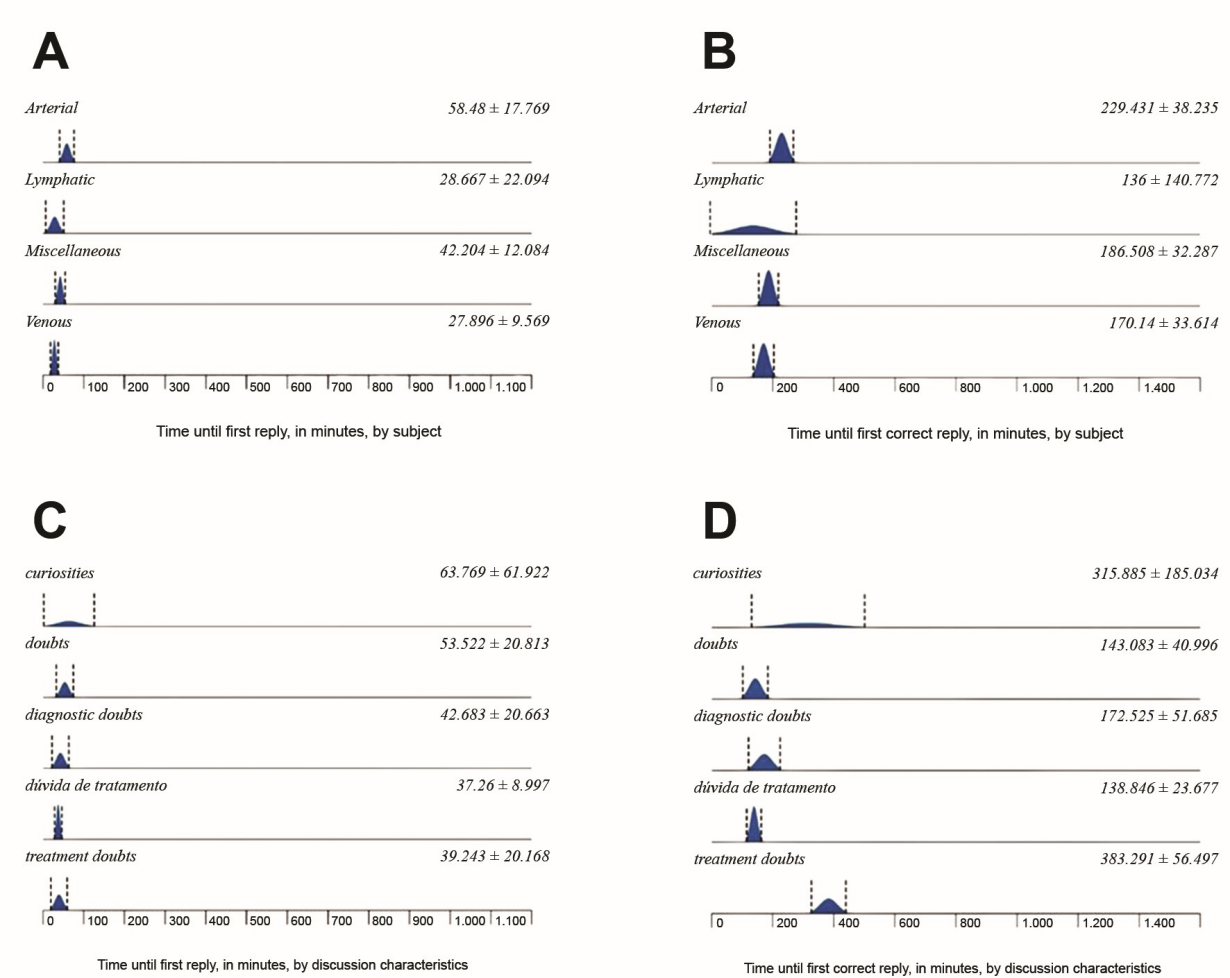
Mean time until first reply and first correct reply by discussion subject (A and B) and characteristics (C and D).

 The distribution of the variables collected across the different subjects discussed can be observed in Tables 1 , 2 and 3 . With relation to response times, the first reply in the arterial subjects category was posted after a mean time of 58.48 ± 17.76 min and the first correct reply was posted at 229.43 ± 38.24 min; in the lymphatic subjects category, times were 28.66 ± 22.09 min for first reply and 136.00 ± 140.77 min for first correct reply; in the venous subject category, they were 27.89 ± 9.56 min for first reply and 170.14 ± 33.614 min for first correct reply; and the first reply to miscellaneous subjects was posted after 42.20 ± 12.08 min while the first correct reply was posted after 186.51 ± 32.28 min ( Figure 3 ). When analyzed by discussion characteristics, diagnostic doubts were answered after 42.68 ± 20.66 min and correctly answered after 172.52 ± 51.68 min; and the same data for other characteristics were, respectively, 63.76 ± 61.92 min and 315.88 ± 185.03 min for curiosities; 37.26 ± 8.99 min and 138.84 ± 23.66 min for treatment doubts, 53.52 ± 20.81 min and 143.08 ± 40.99 min for general doubts; and 39.24 ± 20.16 min and 383.29 ± 56.49 min for case reports (chi-square, p = 0.0094 for time until first reply and p = 0.0587 for time until first correct reply). 

**Table 1:** Distribution of subjects and characteristics of discussions, by number of discussions.

**Subject**	**Discussions (n)**	**Discussions (% of total)**	**Discussions (% of subject)**
Arterial	304	30.01	30.01
Curiosities	2	0.20	0.66
Doubts	22	2.17	7.24
Diagnostic doubts	26	2.57	8.55
Treatment doubts	176	17.37	57.89
Case reports	78	7.70	25.66
Lymphatic	15	1.48	1.48
Doubts	2	0.20	13.33
Diagnostic doubts	3	0.30	20.00
Treatment doubts	7	0.69	46.67
Case reports	3	0.30	20.00
Miscellaneous	358	35.34	35.34
Curiosities	19	1.88	5.31
Doubts	117	11.55	32.68
Diagnostic doubts	48	4.74	13.41
Treatment doubts	120	11.85	33.52
Case reports	54	5.33	15.08
Venous	336	33.17	33.17
Curiosities	5	0.49	1.49
Doubts	64	6.32	19.05
Diagnostic doubts	24	2.37	7.14
Treatment doubts	189	18.66	56.25
Case reports	54	5.33	16.07
Total	1,013	100.00	100.00

**Table 2:** Distribution of subjects and characteristics of discussions, by number of participants.

**Subject**	**Participants (n)**	**Participants (% total)**
Arterial	1,479	29.87
Curiosities	3	0.20
Doubts	70	2.18
Diagnostic doubts	152	2.57
Treatment doubts	943	17.41
Case reports	311	7.52
Lymphatic	76	1.48
Doubts	7	0.20
Diagnostic doubts	15	0.30
Treatment doubts	34	0.69
Case reports	20	0.30
Miscellaneous	1,780	35.41
Curiosities	66	1.88
Doubts	619	11.57
Diagnostic doubts	219	4.75
Treatment doubts	630	11.87
Case reports	246	5.34
Venous	1,862	33.23
Curiosities	24	0.49
Doubts	383	6.33
Diagnostic doubts	138	2.37
Treatment doubts	1,051	18.69
Case reports	265	5.34
Total	5,197	100.00

**Table 3:** Distribution of subjects and characteristics of discussions, by number and type of replies.

**Subject**	**Replies (n)**	**Replies (% of total)**	**Mean time (min) until 1st reply**	**Mean time (min) until 1st correct reply**	**Photos (n)**	**Test results (n)**	**Treatments (n)**
Arterial	4,103	25.67	58.48	229.43	90	165	225
Curiosities	6	0.04	85.50	937.50	0	1	1
Doubts	203	1.27	134.32	175.09	2	0	12
Diagnostic doubts	434	2.72	42.50	151.62	14	12	16
Treatment doubts	2,650	16.58	51.63	170.82	46	96	136
Case reports	810	5.07	57.19	348.79	28	56	60
Lymphatic	228	1.43	28.67	136.00	12	1	10
Doubts	36	0.23	48.00	514.50	0	0	0
Diagnostic doubts	35	0.22	17.33	71.67	3	0	2
Treatment doubts	115	0.72	37.14	61.71	6	0	6
Case reports	42	0.26	7.33	121.33	3	1	2
Miscellaneous	5,433	33.99	42.20	186.51	140	77	118
Curiosities	109	0.68	54.00	303.16	4	0	1
Doubts	2,116	13.24	43.08	137.86	7	3	13
Diagnostic doubts	575	3.60	51.29	145.21	38	13	12
Treatment doubts	1,927	12.06	46.49	130.09	52	38	57
Case reports	706	4.42	18.56	412.94	39	23	35
Venous	6,221	38.92	27.90	170.14	143	110	234
Curiosities	57	0.36	92.20	115.60	2	1	1
Doubts	1,521	9.52	45.02	130.02	12	4	32
Diagnostic doubts	312	1.95	28.83	262.42	18	5	8
Treatment doubts	3,374	21.11	18.03	117.49	82	70	153
Case reports	957	5.99	35.78	366.02	29	30	40
Total	15,985	100.00	42.14	193.21	385	353	587

## DISCUSSION

 One of the most striking changes in behavior over recent decades has occurred in internet-based communication, leading to greater social interaction without borders, 8 and transmission of knowledge has developed in parallel with technological advances. Communication has always played a fundamental role in learning, since, according to Vygotsky, knowledge is constructed within relationships between people, enabling mental development of individuals. 9

 The quest for knowledge is channeled through each person’s experience, generating knowledge, which is improved by what we learn over the course of our lives. By interacting with other people who have had other experiences of the same subjects, we can assimilate and develop new knowledge about the subject, acquiring it collectively. 10 Thus, a group of learners can generate new points of view and new knowledge in conjunction, 11 ^,^ 12 which is a different form of acquisition and transmission of knowledge. 13

 This phenomenon has been observed in social media, where social interaction enables acquisition, storage, transmission, manipulation, and use of information for the purpose of making decisions and judgments. 14 These characteristics were also observed in the discussion group studied here. It could be observed that, even though the group’s members began their medical activities early and finished late, they were always available to participate, whether raising doubts or giving opinions, at any time of day. This contributed to the group accumulating a large quantity of information, with more than 15 thousand replies. 

 Development of communication media is exemplified by the internet, where large quantities of information are generated and stored, creating a virtual library. Adoption of smartphones has facilitated access to and transmission of this information, increasing the quantity of data available for expanding knowledge. However, much unnecessary and incorrect information is also generated, and it is left to each user to filter it for themselves. Creation of groups to discuss specific subjects, irrespective of the platform used, is founded on a common objective and is fundamentally dependent on the capacity of the individual members of the group to interact. 2 Through this relationship, they can produce, exchange, and utilize individual knowledge to construct an idea with a common meaning, thereby achieving greater objectivity and contributing to improving the quality of information. 15

 In the discussion group analyzed here, the largest category of discussions was those related to dealing with doubts (48.6%), showing that this resource is primarily used to help with treatment, after diagnosis has been confirmed, since diagnostic doubts were observed in just 10% of the cases raised. The first replies were posted most quickly to discussions with venous and lymphatic subjects, and the first correct replies were posted earliest to lymphatic and then venous subjects, in that order. The confidence interval was wide for the lymphatic subjects, which is possibly because of the low number of cases discussed. A willingness to help colleagues is evident from the short delay before the first reply, although it takes longer before the correct solution is posted. 

 It has been observed that the relationship between individual intelligence and collective intelligence is weak, demonstrating that it is not necessary that only intelligent people take part in groups, but that it is more important how participants relate to each other within the group. 15 Therefore, incorrect suggestions are also part of the process of improving knowledge, since collective intelligence is influenced by the diversity of the individual people involved. There is a risk of poor results, if a collaborative process is restricted to agreement and consensual tendencies. Surowiecki 16 claims that diversity and independence are important because the best collective decisions are fruit of disagreement and argument, not of consensus or agreement. He defines four conditions that must be taken into account when attributing wisdom to groups: diversity of opinions, in which each individual should have a personal intellectual experience, even if it is only a personal interpretation; independence, so that differing opinions are not allowed to determine one’s own opinion; decentralization; and aggregation, in order to unite the personal contributions to arrive at a collective decision. 16

 These data, evaluated as a whole, lead to the suggestion that group members should not base their conduct on the earliest and quickest replies, but should wait for the subject to mature, which takes an average of 193.21 min. This may seem like a long time for those who are seeking an immediate solution, but for those who are seeking to discuss a case at a clinical meeting or a congress and can therefore wait weeks, it is a very rapid process. 

 The desire to help each other with conduct decisions contributed to formation of a virtual community, constructed on the basis of affinities of interests. Within this community, a process of cooperation developed, 17 and new public knowledge emerges and is shared. 18

 The concept of collective intelligence was created during debates on the subject of technologies of intelligence held by Pierre Lévy. It is characterized by a new form of sustainable thinking via social connections, made possible through use of the open computing networks of the internet. 1 In two studies with 699 people working in groups, convergent evidence was found for a general factor of collective intelligence that can explain the performance of a group. This is strongly correlated with the way in which professionals perceive the emotions of other members of the group (social sensitivity), with the equality of distribution of opinions, and with the proportion of women in the group, because they find it easier to deal with other people’s emotions. 19 In contrast to Artificial Intelligence, the objective of which is to create intelligent machines to substitute people, the objective of collective intelligence is to make people more intelligent. 19

 Intelligence technologies are particularly represented by languages, sign systems, logical resources, and the instruments we make use of. All of our intellectual functioning is induced by these representations. According to Pierre Lévy, the philosopher and sociologist who created the concept of collective intelligence, human beings are incapable of thinking alone without the aid of any kind of tool. 1

 Faced with an elevated incidence of doubts related to clinical cases, the Vascular Forum proved to be a tool of great clinical utility to its participants, confirming the indirect perception of the instrument’s users of the importance of collective intelligence in medicine. This study opens the way for further studies that could assess the correct responses’ evidence levels, thereby measuring the degree of correctness achieved by collective intelligence in discussion groups for solving clinical cases. 

 Collective intelligence is a way for people to think better and share their knowledge with other people. This practice is employed in its written form through books, but with utilization of mechanical resources, such as the internet, it is possible to increase connectivity and transmit knowledge more quickly. Thus, the users themselves create content by interacting with websites. 1

## CONCLUSIONS

 Interaction between specialists using instant messaging technology proved capable of initiating discussions and raising different approaches rapidly. The rate of resolution, considering the time elapsed before the first correct reply was posted, was also high. 
