# Supplementary material for: Vascular Forum: collective intelligence in the resolution of vascular clinical cases
Source: J Vasc Bras. 2018 Jul-Sep;17(3):193–200. doi: 10.1590/1677-5449.005018 (PMC6326134; doi:10.1590/1677-5449.005018)
Supplement: Translation

# Translation: Fórum Vascular: inteligência coletiva na resolução de casos clínicos vasculares

**Authors:** Fabiano Luiz Erzinger, Walter Junior Boim de Araujo, Amanda Ayako Minemura Ordinola, André Felipe Gasparini, André Vítor Timóteo da Luz, Daniela Midori Kamada, Alexandre Campos Moraes Amato

**Affiliations:** 1 Instituto da Circulação, Curitiba, PR, Brasil.; 2 Universidade Santo Amaro – UNISA, São Paulo, SP, Brasil.

**Keywords:** inteligência, fóruns de discussão, informática médica, educação médica

## Abstract

**Contexto:**

A inteligência coletiva tem extrema importância em grupos coletivos de discussão de casos clínicos médicos, auxiliando tanto os profissionais na pronta tomada de decisão como seus pacientes.

**Objetivos:**

Avaliar a resolutividade e as características das discussões clínicas realizadas em grupo fechado de mensagens instantâneas e sua aplicabilidade clínica.

**Métodos:**

Análise retrospectiva de discussões clínicas e eventos no Fórum Vascular, grupo aberto para especialistas em dispositivos móveis.

**Resultados:**

No período de julho de 2015 a julho de 2017, foram discutidos 1.013 assuntos, com 680 membros. Desses assuntos, 26 (2,57%) foram curiosidades, 101 (9,97%) foram dúvidas de diagnóstico, 492 (48,57%) foram dúvidas de tratamento, 205 (20,24%) foram dúvidas gerais e 189 (17,5%) foram relatos de casos. O número médio de interações por evento foi de 16,599 (±1,366). O tempo médio para a primeira resposta foi de 42,14 min (±7,55). Os assuntos discutidos foram 358 casos com miscelânea, 336 casos venosos, 15 casos linfáticos e 304 casos arteriais, totalizando 15.985 respostas.

**Conclusões:**

A interação entre especialistas utilizando a tecnologia de troca de mensagens instantâneas mostrou-se capaz de levantar discussões e abordagens rapidamente. A resolutividade, considerada o tempo da primeira resposta correta, também foi alta. O Fórum Vascular evidenciou-se uma ferramenta de grande valia clínica para seus participantes, confirmando a importância da inteligência coletiva na área médica.

## INTRODUÇÃO

 A inteligência coletiva tem como princípios reconhecer que cada ser humano tem algum conhecimento, porém não em sua totalidade, e considerar as experiências vividas ao longo da vida e que podem ser partilhadas. No contexto da saúde, esse conceito adquire extrema importância em grupos coletivos de resolução de casos clínicos médicos, auxiliando tanto os médicos na pronta tomada de decisão como, consequentemente, os pacientes dependentes de uma medicina de excelência 1 . 

 Desde a década de 1990 2 ^,^ 3 , grupos de especialidades médicas têm sido criados e difundidos, recorrendo às tecnologias de mídias sociais e mensagens instantâneas largamente utilizadas para fins cotidianos. A troca de experiências e a facilidade de acesso à informação têm se mostrado de grande valia, principalmente longe dos grandes centros de informação. 

 A telemedicina formal apresentou importante evolução e consolidação no Brasil nos últimos anos 4 , mas o avanço e a popularização das tecnologias permitiu o crescimento de iniciativas informais, como as redes de aprendizagem colaborativas. Elas utilizam tecnologias interativas para ampliar as possibilidades de construção de conhecimentos, seja aumentando as facilidades de acesso a materiais educacionais de qualidade, seja permitindo o acesso a centros ou colegas de referência ou a estruturação de novas sistemáticas educacionais 4 . 

 Entre as iniciativas que promovem educação permanente para os profissionais de saúde visando a qualificação profissional, a teleducação interativa pode ser um importante recurso para disponibilizar materiais e unidades educacionais de qualidade. Embora grande parte do desenvolvimento educacional seja focada em cursos, a integração com a prática profissional é fundamental para a motivação dos profissionais. Assim, a utilização de métodos de segunda opinião formativa especializada pode ser importante, pois permite desenvolver uma estratégia educacional com foco no aprendizado baseado na problemática real 3 . 

 Após o início informal de grupo de especialistas em aplicativo seguro de troca de mensagens instantâneas, houve a necessidade de expandir a iniciativa com a criação de novos grupos temáticos para possibilitar a acomodação de mais especialistas interessados. Experiências anteriores 2 com outros meios de comunicação mostraram rápida adoção da tecnologia e prontidão em participar, permitindo uma discussão produtiva de assuntos polêmicos apesar da distância geográfica entre os participantes, com grupos menores de 24 participantes. Com o crescimento rápido dos grupos e a constante troca de informações, houve necessidade de quantificar a qualidade e o impacto dessa iniciativa na atuação profissional 5 . Até o presente momento, não há avaliação de grupos vasculares em aplicativos de mensagens instantâneas na literatura. 

 O presente estudo tem como objetivo avaliar a eficácia e a eficiência na análise de casos clínicos em um grupo de discussões de mensagens instantâneas, por meio do tempo e número de respostas. 

## MÉTODOS

 Foi realizado um levantamento retrospectivo observacional dos casos discutidos entre cirurgiões vasculares em um grupo aberto de discussões de mensagens instantâneas com 680 membros, no período de julho de 2015 a julho de 2017. O trabalho foi aprovado pelo Comitê de Ética da instituição com protocolo nº 00012017. 

 Os dados do fórum foram compartilhados entre os membros dos casos clínicos utilizando uma plataforma de mensagens instantâneas (WhatsApp), sendo extraídos por meio de *software* especializado (Whatsapp Pocket) 6 , exportados e convertidos para formato de documento comum. A seguir, foram analisados e tabulados em planilha colaborativa *online* segura (GoogleDocs®). 

 Foi realizada a estratificação dos seguintes dados de cada caso clínico: médico que iniciou a discussão, assunto, data, hora de início, momento da primeira resposta correta, número de respostas, características da discussão (dúvida de tratamento, dúvida diagnóstica, curiosidade, dúvida geral, relato de caso), características do relato (resumo), presença de informações extras no relato (imagem, exames, texto e tratamento) e diagnóstico definitivo. A resposta correta foi considerada a de maior consenso entre os participantes da discussão. 

 Após a verificação manual da consistência dos dados, procedeu-se à análise descritiva e estatística. Para as características expressas como dados categóricos, foram apresentadas as frequências absolutas e as proporções. Na análise estatística, utilizou-se o teste *t*, o qui-quadrado e a análise de variância (ANOVA). A análise estatística multivariada dos dados foi efetuada utilizando o *software* Excel (Microsoft) e Wizard 1.9.20 (Evan Miller). Para as características expressas como dados quantitativos, foram calculados a média e o desvio padrão e apresentados os valores mínimo e máximo 7 . 

## RESULTADOS

 No período de julho de 2015 a julho de 2017, foram discutidos 1.013 assuntos diversos no grupo de troca de mensagens instantâneas. Desses, 26 (2,57%) foram curiosidades, 101 (9,97%) foram dúvidas de diagnóstico, 492 (48,57%) foram dúvidas de tratamento, 205 (20,24%) foram dúvidas gerais e 189 (17,5%) foram relatos de casos ( Figura 1 ). O número médio de interações por evento foi de 16,6 ± 1,37. O tempo médio para a primeira resposta foi de 42,14 ± 7,55 min, enquanto o tempo médio para a primeira resposta correta, considerada a de maior consenso, foi de 193,21 ± 19,73 min. Dessas discussões, 385 (38%) incluem fotos, 353 (34,8%) incluem exames subsidiários, e 426 (42,1%) incluem o tratamento. Com relação à distribuição das horas, o grupo mostrou-se ativo principalmente das 8 h às 22 h ( Figura 2 ). 

**Figura 1:** Distribuição da característica das discussões (A) e dos assuntos (B).

**Figura 2:** Número de discussões por hora do dia (A) e taxa de chegada por hora (B).

 Com relação aos assuntos discutidos, miscelânea liderou com 358 casos (35,3%) e a participação de 5,15 ± 0,47 integrantes do grupo. Quanto a assuntos venosos, foram 336 relatos (33,2%), com a participação de 5,86 ± 0,45 integrantes. Quanto a assuntos linfáticos, foram 15 casos (1,5%), com a participação de 5,43 ± 1,58 integrantes. Quanto a assuntos arteriais, foram 304 casos (30%), com a participação de 5,19 ± 0,44 integrantes (ANOVA, p = 0,1133). 

 Avaliando o número de participantes das discussões com relação à característica, 5,56 ± 0,75 integrantes do grupo participaram de dúvidas em geral, 4,87 ± 0,57 de relatos de caso, 3,72 ± 1,92 de curiosidades, 5,41 ± 0,71 de dúvidas de diagnóstico e 5,61 ± 0,64 de dúvidas de tratamento (ANOVA, p = 0,0728). Quanto ao número de respostas, nos casos de dúvidas de diagnóstico houve 13.979 ± 2.586 respostas, nas dúvidas de tratamento, 17.06 ± 1.70 respostas, nas curiosidades, 6,88 ± 3,65 respostas, nas dúvidas em geral, 19,98 ± 4,28 respostas, e nos relatos de caso, 14,45 ± 3,27 respostas (ANOVA, p = 0,0103). 

 A distribuição das variáveis coletadas nos assuntos discutidos pode ser observada nas Tabelas 1 , 2 e 3 . Com relação às respostas, no assunto arterial, a primeira resposta ocorreu com tempo médio de 58,48 ± 17,76 min e a primeira resposta correta surgiu com 229,43 ± 38,24 min; no assunto linfático, 28,66 ± 22,09 min para primeira resposta e 136.00 ± 140,77 min para primeira resposta correta; no assunto venoso, 27,89 ± 9,56 min para primeira resposta e 170,14 ± 33,614 min para primeira resposta correta; e no assunto miscelânea, 42,20 ± 12,08 min para primeira resposta e 186,51 ± 32,28 min para primeira resposta correta ( Figura 3 ). Pelas características da discussão, as dúvidas diagnósticas foram respondidas em 42,68 ± 20,66 min e corretamente em 172,52 ± 51,68 min; curiosidades em 63,76 ± 61,92 min e corretamente em 315,88 ± 185,03 min; dúvidas de tratamento em 37,26 ± 8,99 min e corretamente em 138,84 ± 23,66 min; dúvidas gerais em 53,52 ± 20,81 min e corretamente em 143,08 ± 40,99 min; e relatos de casos em 39,24 ± 20,16 min e corretamente em 383,29 ± 56,49 min (qui-quadrado, p = 0,0094 para tempo de resposta e p = 0,0587 para tempo de primeira resposta correta). 

**Tabela 1:** Distribuição dos assuntos e das características da discussão de acordo com o número de discussões.

**Assunto**	**Discussões (n)**	**Discussões (% total geral)**	**Discussões (% assunto)**
Arterial	304	30,01	30,01
Curiosidade	2	0,20	0,66
Dúvida	22	2,17	7,24
Dúvida diagnóstica	26	2,57	8,55
Dúvida de tratamento	176	17,37	57,89
Relato de caso	78	7,70	25,66
Linfático	15	1,48	1,48
Dúvida	2	0,20	13,33
Dúvida diagnóstica	3	0,30	20,00
Dúvida de tratamento	7	0,69	46,67
Relato de caso	3	0,30	20,00
Miscelânea	358	35,34	35,34
Curiosidade	19	1,88	5,31
Dúvida	117	11,55	32,68
Dúvida diagnóstica	48	4,74	13,41
Dúvida de tratamento	120	11,85	33,52
Relato de caso	54	5,33	15,08
Venoso	336	33,17	33,17
Curiosidade	5	0,49	1,49
Dúvida	64	6,32	19,05
Dúvida diagnóstica	24	2,37	7,14
Dúvida de tratamento	189	18,66	56,25
Relato de caso	54	5,33	16,07
Total geral	1.013	100,00	100,00

**Tabela 2:** Distribuição dos assuntos e das características da discussão de acordo com o número de participantes.

**Assunto**	**Participantes (n)**	**Participantes (% total geral)**
Arterial	1.479	29,87
Curiosidade	3	0,20
Dúvida	70	2,18
Dúvida diagnóstica	152	2,57
Dúvida de tratamento	943	17,41
Relato de caso	311	7,52
Linfático	76	1,48
Dúvida	7	0,20
Dúvida diagnóstica	15	0,30
Dúvida de tratamento	34	0,69
Relato de caso	20	0,30
Miscelânea	1.780	35,41
Curiosidade	66	1,88
Dúvida	619	11,57
Dúvida diagnóstica	219	4,75
Dúvida de tratamento	630	11,87
Relato de caso	246	5,34
Venoso	1.862	33,23
Curiosidade	24	0,49
Dúvida	383	6,33
Dúvida diagnóstica	138	2,37
Dúvida de tratamento	1.051	18,69
Relato de caso	265	5,34
Total geral	5.197	100,00

**Tabela 3:** Distribuição dos assuntos e das características da discussão de acordo com o número e o tipo de respostas.

**Assunto**	**Respostas (n)**	**Respostas (% total geral)**	**Média de tempo (min) para 1ª resposta**	**Média de tempo (min) para 1ª resposta correta**	**Fotos (n)**	**Exames (n)**	**Tratamentos (n)**
Arterial	4.103	25,67	58,48	229,43	90	165	225
Curiosidade	6	0,04	85,50	937,50	0	1	1
Dúvida	203	1,27	134,32	175,09	2	0	12
Dúvida diagnóstica	434	2,72	42,50	151,62	14	12	16
Dúvida de tratamento	2.650	16,58	51,63	170,82	46	96	136
Relato de caso	810	5,07	57,19	348,79	28	56	60
Linfático	228	1,43	28,67	136,00	12	1	10
Dúvida	36	0,23	48,00	514,50	0	0	0
Dúvida diagnóstica	35	0,22	17,33	71,67	3	0	2
Dúvida de tratamento	115	0,72	37,14	61,71	6	0	6
Relato de caso	42	0,26	7,33	121,33	3	1	2
Miscelânea	5.433	33,99	42,20	186,51	140	77	118
Curiosidade	109	0,68	54,00	303,16	4	0	1
Dúvida	2.116	13,24	43,08	137,86	7	3	13
Dúvida diagnóstica	575	3,60	51,29	145,21	38	13	12
Dúvida de tratamento	1.927	12,06	46,49	130,09	52	38	57
Relato de caso	706	4,42	18,56	412,94	39	23	35
Venoso	6.221	38,92	27,90	170,14	143	110	234
Curiosidade	57	0,36	92,20	115,60	2	1	1
Dúvida	1.521	9,52	45,02	130,02	12	4	32
Dúvida diagnóstica	312	1,95	28,83	262,42	18	5	8
Dúvida de tratamento	3.374	21,11	18,03	117,49	82	70	153
Relato de caso	957	5,99	35,78	366,02	29	30	40
Total geral	15.985	100,00	42,14	193,21	385	353	587

**Figura 3:** Tempo da primeira resposta e da primeira resposta correta por assunto (A e B) e característica da discussão (C e D).

## DISCUSSÃO

 Entre as mudanças mais notáveis de comportamento nas últimas décadas está a comunicação baseada na internet, que acarretou uma maior interação social sem fronteiras 8 , assim como a transmissão do conhecimento tem evoluído em paralelo ao avanço da tecnologia. A comunicação sempre exerceu grande participação no aprendizado, pois, segundo Vygotsky, é na relação entre as pessoas que o conhecimento é construído, permitindo o desenvolvimento mental do indivíduo 9 . 

 A busca por conhecimento passa pela vivência de cada pessoa e gera sabedoria, que é aprimorada com aquilo que aprendemos ao longo da vida. Ao interagirmos com outras pessoas, que tiveram outras vivências relacionadas ao mesmo tema, podemos assimilar e desenvolver um novo conhecimento sobre aquele assunto, adquirido coletivamente 10 . Assim, um grupo de aprendizes pode desenvolver conjuntamente novos pontos de vistas e conhecimentos 11 ^,^ 12 , o que representa outra forma de aquisição e transmissão do conhecimento 13 . 

 Tal fato tem sido observado nas mídias sociais, onde a interação social permite a aquisição, armazenamento, transmissão, manipulação e uso de informações para fins de decisões e julgamentos 14 . Da mesma forma, tais características também foram observadas no grupo de discussão em estudo. Pôde-se perceber que, apesar de seus integrantes iniciarem suas atividades médicas cedo e terminarem tarde, sempre estavam disponíveis para participar, fosse apresentando dúvidas ou dando opiniões, a qualquer hora do dia. Isso contribuiu para a geração de um grande montante de informações, com mais 15 mil respostas. 

 O avanço das formas de comunicação é evidenciado na internet, onde é gerada e armazenada uma grande quantidade de informações, formando uma biblioteca virtual. Com o uso de *smartphones*, o acesso e a transmissão dessas informações foram facilitados, gerando um maior número de dados para melhorar o conhecimento. No entanto, são geradas muitas informações desnecessárias e incorretas, cabendo a cada um a filtragem delas. A criação de grupos para discussões de assuntos específicos, independentemente da plataforma, possui um propósito comum, que depende fundamentalmente da capacidade dos indivíduos e do grupo de interagirem 2 . Dessa relação, eles podem produzir, trocar e utilizar o conhecimento individual para construir uma ideia de senso comum, sendo assim mais objetivos e contribuindo para a melhoria da qualidade das informações 15 . 

 No grupo de discussão analisado, a maior quantidade de assuntos foi relacionada a dúvidas de tratamento (48,6%), evidenciando que o recurso é utilizado principalmente para auxílio ao tratamento, após o diagnóstico já estar firmado, pois as dúvidas de diagnóstico ocorreram em apenas 10% dos casos apresentados. Houve maior velocidade na primeira resposta em assuntos venosos e linfáticos, e na primeira resposta correta em assuntos linfáticos e venosos, nessa ordem. O intervalo de confiança em assuntos linfáticos foi grande, possivelmente pela pequena quantidade de casos apresentados. A prontidão em ajudar o colega é evidente nos baixos tempos de primeira resposta, porém a qualificação da resposta requer tempo maior. 

 Tem-se observado uma fraca relação entre inteligência individual e inteligência coletiva, demonstrando que, nos grupos, não há a necessidade da participação somente de pessoas inteligentes, sendo mais importante a maneira como os participantes se relacionam dentro do grupo 15 . Portanto, fazem parte do processo de melhorar o conhecimento as respostas erradas, pois a inteligência coletiva é influenciada pela diversidade dos indivíduos envolvidos. Em um processo colaborativo, não pode haver somente concordâncias e tendências consensuais, correndo-se o risco de obtenção de resultados mais pobres. Surowiecki 16 afirma que a diversidade e a independência são importantes porque as melhores decisões coletivas são fruto de discordância e contestação, não de consenso ou acordo. O autor define quatro condições necessárias que devem ser levadas em conta ao atribuir a sabedoria aos coletivos: a diversidade de opiniões, em que cada indivíduo deve possuir uma vivência intelectual pessoal, nem que seja apenas uma interpretação individual; a independência, de forma a não deixar que as opiniões diversas determinem a sua opinião própria; a descentralização; e a agregação, na tentativa de unir as contribuições pessoais no sentido de chegar a uma decisão coletiva 16 . 

 A partir desses dados, quando avaliados globalmente, sugere-se para os membros do grupo que não baseiem sua conduta nas primeiras respostas rápidas, mas esperem o assunto maturar, em média, 193,21 min. Esse tempo pode parecer alto para aqueles que buscam uma resposta imediata, mas para aqueles que buscam a discussão de um caso em uma reunião clínica ou congresso e podem, portanto, esperar semanas é muito rápido. 

 A preocupação em auxiliar as condutas contribui para a formação de uma comunidade virtual, construída com base em afinidades e interesses. Nela, desenvolve-se um processo de cooperação 17 , e novos conhecimentos públicos surgem e são compartilhados 18 . 

 O conceito de inteligência coletiva foi criado a partir de alguns debates realizados por Pierre Lévy relacionados às tecnologias da inteligência. Caracteriza-se pela nova forma de pensamento sustentável através de conexões sociais que se tornam viáveis pela utilização das redes abertas de computação da internet 1 . Em dois estudos com 699 pessoas, trabalhando em grupos, encontraram-se evidências convergentes de um fator geral de inteligência coletiva que explica o desempenho de um grupo. Este está fortemente correlacionado à maneira como os profissionais percebem as emoções dos outros membros do grupo (sensibilidade social), à igualdade na distribuição das opiniões e à proporção de mulheres no grupo, pois elas têm maior facilidade de lidar com as emoções de outras pessoas 19 . Ao contrário da inteligência artificial, na qual procura-se criar máquinas inteligentes para substituir as pessoas, a inteligência coletiva procura deixar as pessoas mais inteligentes 19 . 

 As tecnologias da inteligência são representadas especialmente pelas linguagens, sistemas de sinais, recursos lógicos e instrumentos dos quais nos servimos. Todo o nosso funcionamento intelectual é induzido por essas representações. Segundo o filósofo e sociólogo criador do conceito de inteligência coletiva, Pierre Lévy, os seres humanos são incapazes de pensar sós e sem o auxílio de qualquer ferramenta 1 . 

 Devido à grande incidência de dúvidas em casos clínicos, o Fórum Vascular mostrou-se uma ferramenta de grande valia clínica para seus participantes, confirmando a percepção indireta dos usuários do instrumento da importância da inteligência coletiva na área médica. Este estudo abre caminho para novas pesquisas que podem abordar o grau de evidência das respostas corretas e, assim, mensurar a assertividade da inteligência coletiva em grupos de discussão na resolução de casos clínicos. 

 A inteligência coletiva é uma forma de o homem pensar e compartilhar melhor seus conhecimentos com outras pessoas. Essa prática é utilizada na forma escrita nos livros, mas com a utilização de recursos mecânicos, como a internet, consegue-se aumentar a conectividade e transmitir de uma maneira mais rápida o conhecimento. Nela, os próprios usuários geram o conteúdo através da interatividade com o *website* 1 . 

## CONCLUSÃO

 A interação entre especialistas utilizando a tecnologia de troca de mensagens instantâneas mostrou-se capaz de levantar discussões e abordagens rapidamente. A resolutividade, considerada o tempo da primeira resposta correta, também foi alta.
